# Evolution and stress-responsive regulation of the apyrase gene family in Chinese cabbage (*Brassica rapa* L. ssp. *pekinensis*): a genome-wide perspective on temperature and drought tolerance

**DOI:** 10.3389/fpls.2026.1726966

**Published:** 2026-07-29

**Authors:** Yang Zhou, Jiangtao Du, Shanyu Li, Xinjie Zhang, Yonghui Zhao, Lin Hao, Wei Fu

**Affiliations:** 1 College of Life Science, Shenyang Normal University, Shenyang, China; 2 Institute of Quality Standards & Testing Technology for Agri-Products, Xinjiang Academy of Agricultural Sciences, Urumqi, China

**Keywords:** abiotic stress, apyrase, *Brassica rapa*, evolutionary relationships, gene expression pattern

## Abstract

Apyrases (APYs), nucleoside triphosphate-diphosphohydrolases, serve as critical enzymes in regulating extracellular ATP levels and maintaining biochemical homeostasis under stress conditions. Under the escalating pressures of global climate change, the adverse effects of rising temperatures and altered precipitation patterns on crop productivity have become increasingly severe. Chinese cabbage (*Brassica rapa* L. ssp. *pekinensis*, *B.rapa*), a globally significant cruciferous crop, faces substantial threats due to its high sensitivity to environmental stressors. Throughout its growth cycle, it is frequently subjected to various environmental stresses and exhibits high sensitivity to drought stress and temperature fluctuations. In this study, we systematically identified 13 *APY* genes in the *B.rapa* genome and conducted a comprehensive analysis of their phylogenetic relationships, conserved motifs, and cis-regulatory elements. qRT-PCR analysis further revealed distinct tissue-specific expression patterns of *BrAPYs* and their differential regulation under drought, extreme temperature, and cold stress conditions. These findings establish a molecular framework for understanding the stress-responsive functions of *APYs* in Chinese cabbage and highlight potential targets for enhancing stress tolerance through genetic improvement.

## 1 Introduction

Apyrase (APY) functions as a nucleotide triphosphate diphosphohydrolase, catalyzing the hydrolysis of various nucleotides, including ATP, UTP, ADP, and UDP, into their monophosphate derivatives (AMP/UMP) (Robson et al., 2006). These hydrolysis products undergo extracellular conversion to adenosine and uridine, facilitating nucleotide recycling essential for maintaining cellular homeostasis (Zimmermann, 2021). The energy released by ATP hydrolysis drives critical biochemical processes, including abiotic stress responses (Mohammad-Sidik et al., 2021), wound signaling, and cell expansion (Chivasa et al., 2005). Notably, excessive extracellular ATP (eATP) accumulation exerts growth-inhibitory effects through sustained receptor activation (Roux and Steinebrunner, 2007), a process mitigated by APY-mediated eATP hydrolysis (Clark and Roux, 2018). Currently, while APY functions have been characterized in model species like *Arabidopsis* (Chiu et al., 2015) and crops such as wheat (Liu et al., 2019), their regulatory mechanisms in *Brassica* crops remain underexplored.

*APYs* are initially identified in potato (*Solanum tuberosum*) with seven family members (Riewe et al., 2008), have been extensively characterized across diverse plant taxa. Although present in spore-bearing plants, their functional roles are more thoroughly elucidated in angiosperms (Clark et al., 2021). *APY* genes have been identified in several plant species, such as *Arabidopsis thaliana* (Chiu et al., 2015), wheat (Liu et al., 2019), peanut (Sharif et al., 2023), and rice (Chowdhury et al., 2023). For instance, all seven *APYs* found in *Arabidopsis thaliana* contain five characteristic APY domains. *AtAPY1* and *AtAPY2* have been confirmed to play a critical role in plant sexual reproduction, as evidenced by double mutants of *AtAPY1*/*AtAPY2* exhibiting complete inhibition of pollen germination (Chiu et al., 2015; Steinebrunner et al., 2003). These enzymes regulate extracellular ATP levels, thereby maintaining eATP homeostasis essential for growth regulation (Wu et al., 2007; Lim et al., 2014). Nine *APYs* have been characterized in wheat, and their expressions and enzymatic activities exhibit distinct responses to stress conditions including heavy metals, heat, and salt. For instance, the *TaAPY3–1* demonstrates significantly higher ATP hydrolysis efficiency compared to other isoforms *in vitro* systems (Liu et al., 2019). Among 17 *APY* homologous genes in peanuts, *AhPAY2-1P* specifically activates pathogen-responsive genes in pericarp tissues (Sharif et al., 2023). In rice, all nine *APYs* involve in various stress responses, hormone signaling, and plant development by differential expressions (Chowdhury et al., 2023). Similarly, among 16 *APYs* identified in the maize genome, *ZmAPY5/APY16* genes are significantly associated with drought tolerance and agronomic traits (He et al., 2024). Additionally, four putative APY genes were identified in the model legume *Medicago truncatula*, where they participate in the early stages of nodule formation (Cohn et al., 2001).

Chinese cabbage (*Brassica rapa* L. ssp. *pekinensis*), a cold-tolerant cruciferous vegetable of substantial agronomic importance in Asia (Quan et al., 2022), encounters cultivation challenges such as delayed germination, growth inhibition, and reduced yield under temperature and drought stresses (Kayum et al., 2015). Although adenosine triphosphatases (*APYs*) are established regulators of abiotic stress responses, their functional roles in *B.rapa* remain largely uncharacterized. This study systematically identifies *BrAPYs* at the genome-wide level, elucidating their sequence features, stress-responsive expression profiles, and transcriptional regulatory mechanisms. These findings enhance our understanding of the potential contributions of *BrAPYs* to stress adaptation and provide valuable candidate molecular targets for crop improvement strategies.

## 2 Materials and methods

### 2.1 Identification of *APY* gene family

The protein sequences of seven *Arabidopsis thaliana* APY family members (*AtAPY1*-*AtAPY7*) were retrieved from the TAIR database (https://www.arabidopsis.org/) (Swarbreck et al., 2008). A BLASTP search against the BRAD database (http://www.brassicadb.cn/) (Chen et al., 2022) identified putative *BrAPY* homologs in *B.rapa.* Subsequently, the TBtools software (v2.110) and the Hidden Markov Model (HMMER) were employed for screening. The candidate sequences were uploaded to InterPro (https://www.ebi.ac.uk/interpro/) (Paysan-Lafosse et al., 2023) for analysis to detect the conserved GDA1/CD39 domain, which is a characteristic feature of APY family members. The ACR (Apyrase conserved region) that the genes should possess was determined, and the online tool ESPript 3 (http://espript.ibcp.fr/ESPript/ESPript/) was used for plotting.

### 2.2 Chromosomal distribution and gene structure analysis

The chromosomal distribution of *BrAPYs* was determined based on positional data retrieved from the BRAD database. TBtools (v2.110) was utilized to visualize gene loci and calculate chromosomal density using its integrated Gene Density Profiler module. For structural analysis, coding sequences (CDS) were aligned with their corresponding genomic sequences and further analyzed on the Gene Structure Display Server 2.0 (https://gsds.gao-lab.org/Gsds_help.php) (Hu et al., 2015) to elucidate exon-intron architectures.

### 2.3 Gene replication analysis

Gene duplication events were identified through comparative analysis of genome and the gff file using TBtools (v2.110) (Chen et al., 2023). The non-synonymous (Ka) and synonymous (Ks) substitution rates were calculated via the built-in maximum likelihood algorithm. Duplication time estimation was performed using the molecular clock formula T = Ks/(2λ), with the mutation rate λ set at 1.5×10^-8^ substitutions per site per year (Koch et al., 2000).

### 2.4 Cis-regulatory element analysis

Promoter regions (2,000 bp upstream) of *BrAPYs* were retrieved from the *B.rapa* genome (BRAD database) using EditSeq software. Potential cis-regulatory elements were predicted through analysis with PlantCARE database (https://bioinformatics.psb.ugent.be/webtools/plantcare/html/). The identified elements were systematically classified and visualized using TBtools (v2.110).

### 2.5 Protein characterization

The physicochemical properties of BrAPY proteins, including molecular weight (MW), isoelectric point (pI), and grand average of hydropathicity (GRAVY), were calculated using Expasy - ProtParam (https://web.expasy.org/protparam/). Subcellular localization was predicted using CELLO v2.5 (http://cello.life.nctu.edu.tw/). The protein tertiary structures were modeled using SWISS-MODEL (https://swissmodel.expasy.org/) with default homology modeling parameters (Waterhouse et al., 2018). The Ramachandran Plot, Zlab (https://swissmodel.expasy.org/), was employed to validate the predicted 3D models of the APY proteins (Anderson et al., 2005).

### 2.6 Conserved motif identification

Conserved motifs in BrAPY proteins were identified using MEME Suite v5.3.0 (https://meme-suite.org/meme/) (Bailey et al., 2009), with a maximum motif count set to 10. Functional annotations of the identified motifs were integrated using Pfam domain data (Mistry et al., 2021). The motifs were ranked by detection frequency and mapped to their corresponding BrAPY proteins. Visualize the results using TBtools (v2.110).

### 2.7 Synteny analysis

Comparative genomic analysis between *Arabidopsis thaliana* and *B.rapa* was conducted using the MCScanX module in TBtools. *Arabidopsis thaliana* and *B.rapa* the MCScanX module in TBtools (v2.110). Genome annotation files (GFF3 format) and collinearity data were processed to identify conserved syntenic blocks. When running MCScanX, the analysis parameters are set as follows: 2 CPU threads are used for BLASTP comparison, an E-value cutoff was set at 10^-10^, and the top five BLAST matching results are retained for subsequent homology analysis.

### 2.8 Phylogenetic reconstruction

APY protein sequences from *Arabidopsis thaliana*, rice (*Oryza sativa*), wheat (*Triticum aestivum*), and *B.rapa* were aligned (screening **Supplementary Table 1**). The protein sequences of rice were obtained from Ensemblplants (https://plants.ensembl.org/index.html). For wheat, the nucleic acid sequences were referenced from the wheat genome data (Liu et al., 2019) and the NCBI wheat database, and the protein sequences were derived from the nucleic acid sequences through ORFfinder analysis. A phylogenetic tree was constructed in MEGA11. Based on the results of multiple sequence alignment, the Neighbor-Joining (NJ) clustering algorithm was implemented to construct a phylogenetic tree of the APY gene family. The number of Bootstrap replicates was set to 1000 to evaluate the confidence of branches, and the remaining parameters were maintained at the default settings of the program to ensure the standardization of the analysis process and the reproducibility of the results. The resulting tree diagram was visualized using iTol (https://itol.embl.de/).

### 2.9 Plant cultivation and stress treatments

Eighty seeds of *B.rapa* were surface-sterilized and germinated on moist filter paper at 25°C under dark conditions for 24 h. Germinated seeds were transplanted into a growth substrate mixture (soil:vermiculite:perlite = 3:2:1) and maintained in controlled artificial climate chambers under 12 h light/12 h dark photoperiod at 24°C. At the five-leaf stage, plants were subjected to the following treatments: (1) heat stress (40°C), (2) cold stress (4°C), and (3) drought stress (20% PEG6000 solution). The fourth true leaves were collected at 0, 3, 6 and 12 h post-treatment, and conducted for RNA-seq and qRT-PCR analysis.

### 2.10 qRT-PCR expression analysis

Total RNA was extracted using the Prime Script RT - Reagen kit (TaKaRa) following the kit’s instructions. After RNA extraction, the following reagents were successively added to an RNA - free centrifuge tube: 5.5 μL of nuclease - free water, 2 μL of 5×Primescript buffer, 0.5 μL of complementary deoxynucleotide primers, 0.5 μL of random hexamer primers, 0.5 μL of Prime script RT enzyme mixture, and 1 μL of RNA. The mixture was thoroughly mixed and centrifuged for 30 seconds. Then, the centrifuge tube was placed in a 37 °C water bath for 15 minutes and immediately transferred to an 85 °C water bath for 5 seconds. The above reaction generated cDNA, which was diluted for qRT-PCR analysis. Subsequently, the gene expression levels were quantitatively analyzed on the Lightcycler 96 qRT-PCR platform (Roche, Basel, Switzerland). The gene expression levels were analyzed according to the amplification conditions. Using the amplification conditions as an internal control, the relative expression level of each gene was calculated by the 2^-△△CT^ method. In the qRT-PCR assay, each sample was subjected to 3 technical replicate analyses. The subsequent calculations were based on the average Ct value obtained from the replicate experiments. Specific primers were designed using the NCBI database and synthesized by Shanghai Sangon Biotech Co., Ltd. In the experiment, Actin was used as the internal reference gene, and the primers used for qRT-PCR analysis are listed in **Supplementary Table 2**.

## 3 Results

### 3.1 Identification and analysis of *BrAPYs* in *B.rapa*

The 7 APY protein sequences of *Arabidopsis thaliana* were subjected to BLASTP search in the *B.rapa* gene bank BRAD. Thirteen genes were identified in the genome of *B.rapa*. Interpro sequence analysis indicated that all 13 genes contain the GDA1-CD39 domain. The theoretical isoelectric point (pI) of the BrAPY family varies from 5.38 (BrAPY6-2) to 9.92 (BrAPY7-3), with an average value of 7.942 (**Table 1**). The molecular weight of *BrAPYs* ranges from 24043.10 (BrAPY5) to 107849.91 (BrAPY1-4), averaging at 46976.95. The protein length (AA) ranges from 120 (BrAPY1-5) to 987 (BrAPY1-4). A positive correlation exists between the protein length of and its molecular weight. The mean coefficients of hydrophilicity are all negative, suggesting that they are hydrophilic proteins. Other physicochemical properties and CDS length information are presented in the table. The subcellular localization of proteins was predicted on the CELLO2.5 website, revealing that most of the proteins are located on the plasma membrane, while some are in the nucleus and chloroplast.

**Table 1:** Amino acid composition and physiochemical characteristics of APY proteins in *B.rapa*.

Gene name	Gene ID	Location	AA	MW	pI	Instability index	GRAVY value	SC localization
*BrAPY1-1*	*BraA05g043440.3.5C*	A05(28311455…28315265)-	468	50632.82	7.13	42.43	-0.114	Chloroplast
*BrAPY1-2*	*BraA01g044430.3.5C*	A01(28715076…28720214) +	730	78072.76	7.92	37.27	0.102	Plasma membrane
*BrAPY1-3*	*BraA10g021900.3.5C*	A10(15517031…15521101) +	612	66888.83	5.98	45.58	-0.312	Nuclear
*BrAPY1-4*	*BraA03g008480.3.5C*	A03(3629484…3635114) -	987	107849.91	6.05	41.84	-0.216	Nuclear/Chloroplast/Cytoskeletal
*BrAPY1-5*	*BraA08g024460.3.5C*	A08(17607897…17608919) -	120	13426.28	9.20	53.41	-0.388	Mitochondrial
*BrAPY4-1*	*BraA06g010340.3.5C*	A06(5628099…5631087) +	490	53523.94	8.44	32.08	-0.134	Plasma membrane/Chloroplast
*BrAPY4-2*	*BraA06g010350.3.5C*	A06(5631786…5634348) +	482	53376.73	7.22	36.01	-0.144	Plasma membrane
*BrAPY5*	*BraA07g009220.3.5C*	A07(9033938…9035855) -	220	24043.10	8.73	32.57	-0.300	Nuclear
*BrAPY6-1*	*BraA06g040920.3.5C*	A06(26295701…26298831) +	560	61764.14	8.6	32.55	-0.119	Plasma membrane
*BrAPY6-2*	*BraA09g061270.3.5C*	A09(41921834…41923437) +	283	32505.44	5.38	58.23	-0.715	Nuclear
*BrAPY7-1*	*BraA01g010610.3.5C*	A01 (5449997…5452938) -	734	80967.45	9.57	45.97	-0.232	Plasma membrane
*BrAPY7-2*	*BraA03g049390.3.5C*	A03(24734927…24736278) -	427	47355.07	9.10	48.98	-0.163	Plasma membrane
*BrAPY7-3*	*BraA03g049400.3.5C*	A03(24736321…24737860) -	336	37394.9	9.92	42.57	-0.161	Plasma membrane

### 3.2 Chromosomal localization of the *BrAPYs*

**Figure 1:**
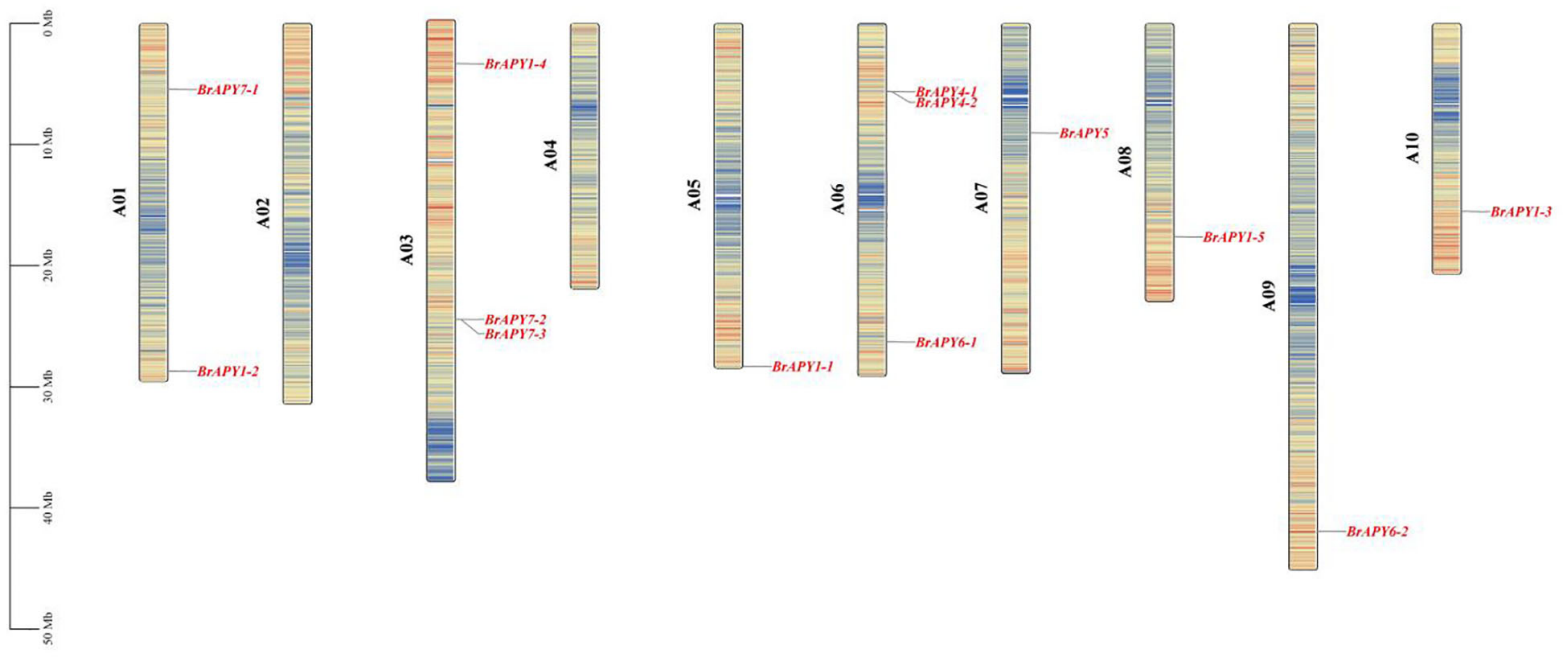
Positions of 13 *BrAPY* genes on chromosomes of Chinese cabbage.

Chromosome distribution analysis showed that the *BrAPY* genes are unevenly distributed across the chromosomes (**Figure 1**). The *BrAPY* genes were named according to their phylogenetic orthology with *Arabidopsis thaliana* apyrases. Specifically, *BrAPY1–2* and *BrAPY7–1* are located on chromosome 1; *BrAPY1-4*, *BrAPY7–2* and *BrAPY7–3* are found on chromosome 3; *BrAPY1–1* resides on chromosome 5; *BrAPY4-1*, *BrAPY4–2* and *BrAPY6–1* are positioned on chromosome 6; *BrAPY5* is situated on chromosome 7; *BrAPY1–5* is on chromosome 8; *BrAPY6–2* is located on chromosome 9; and *BrAPY1–3* is found on chromosome 10.

### 3.3 Analysis of conserved motifs and gene structure

**Figure 2:**
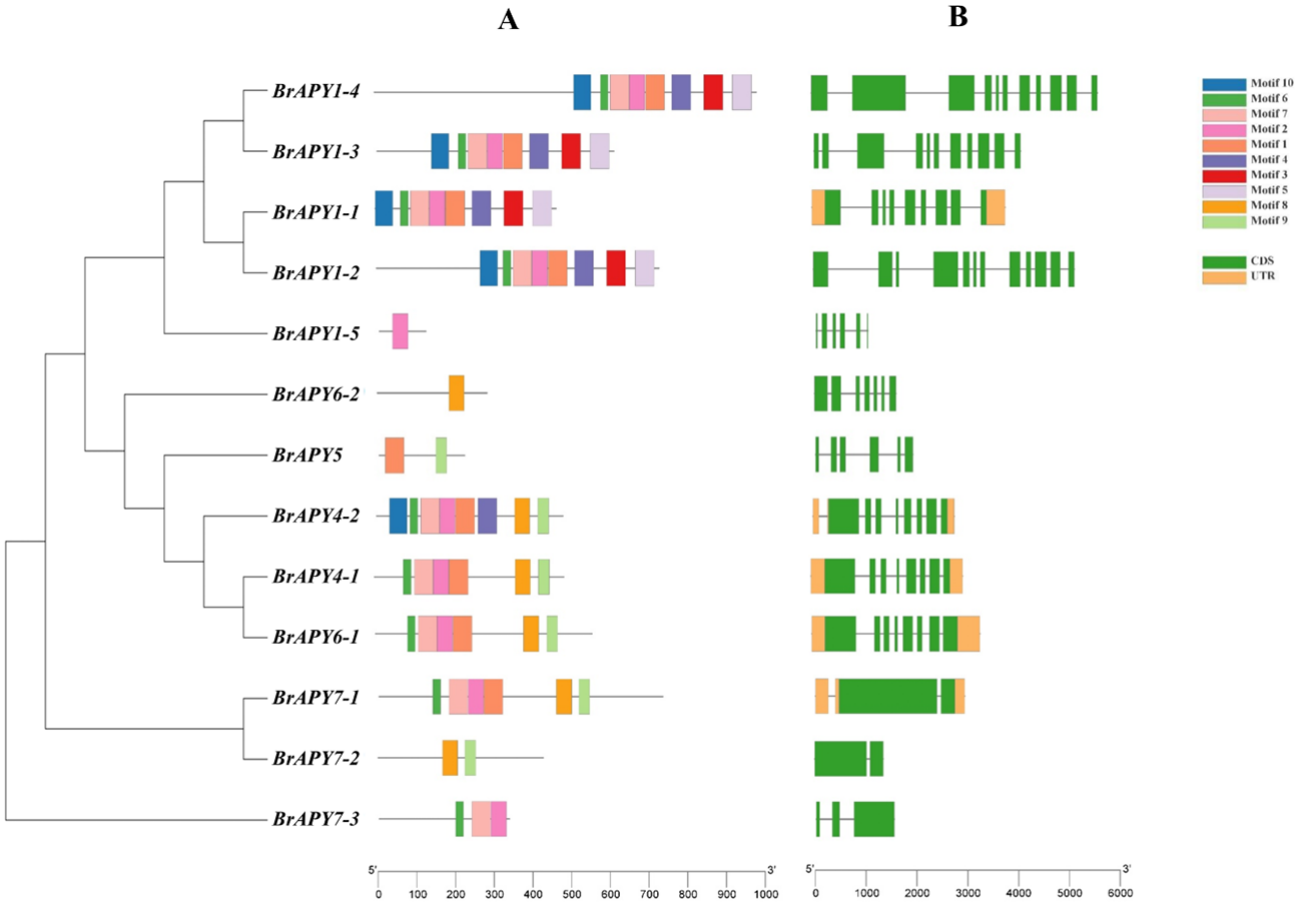
Conserved motifs and gene structure analysis of *BrAPYs*: **(A)** Phylogenetic distribution of conserved motifs predicted by MEME suite analysis. **(B)** Exon-intron organization patterns. The legend is located on the right side of the picture.

Phylogenetic motif analysis of the 13 *BrAPYs* was performed utilizing the MEME Suite (**Figure 2A**). The distribution of conserved motifs exhibited a strong correlation with phylogenetic classification, with nearly identical motif arrangements observed among members of the same clade, except for *BrAPY5*, which displayed distinct patterns. Specifically, Group I predominantly contain eight motifs, with the exception of *BrAPY1-5*, which retains only one motif. In Group II, *BrAPY4-1*, *BrAPY4-2*, and *BrAPY6–1* each exhibits six conserved motifs, contrasting sharply with *BrAPY5* (two motifs) and *BrAPY6-2* (one motif). Group III showed variable motif composition, with *BrAPY7–1* containing five motifs, *BrAPY7–2* two motifs, and *BrAPY7–3* three motifs. A total of ten conserved motifs (numbered 1-10) were identified through systematic (screening **Supplementary Table 3**). Among these, motifs 3, 8, 9, and 10 represent novel uncharacterized domains with potential functional significance, while motifs 1, 2, 4, 5, 6, and 7 correspond to established GDA1/CD39 family signatures. Notably, motif 2, 6, and 7 demonstrate a conservation across all *BrAPYs*.

According to the gene structure map of *BrAPYs*, most *BrAPYs* have different numbers of exons and introns (**Figure 2B**). The coding sequences contain between 2–12 exons (1–11 introns), with distinct architectural patterns emerging across phylogenetic groups. Strikingly, three closely related members of Group II (*BrAPY4-2*, *BrAPY4-1*, and *BrAPY6-1*) shared an identical exon-intron configuration (eight exons and seven introns), suggesting structural conservation within this subgroup.

### 3.4 Gene duplication analysis

**Figure 3:**
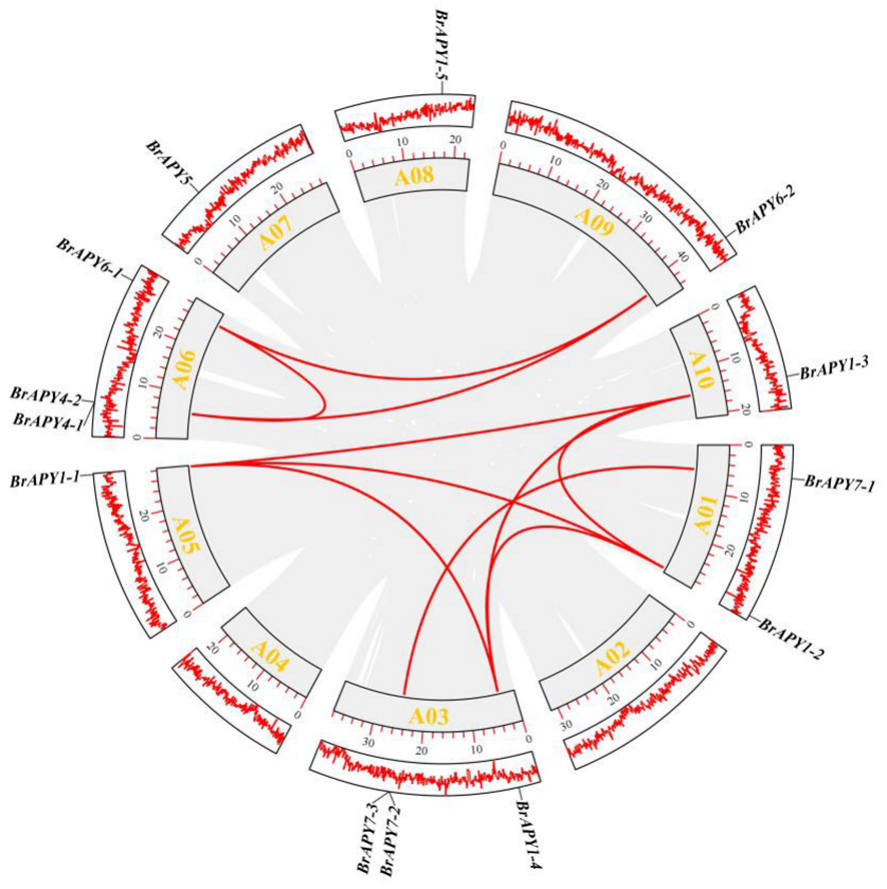
Syntenic relationships of duplicated *BrAPY* gene pairs. Red lines show intra-family duplication events, while gray lines represent conserved syntenic blocks across chromosomes. The outermost red bars indicate chromosome-level gene density.

The *B.rapa* genome had a total of ten duplications, eight of which were in the APY family (**Figure 3**). Ka (nonsynonymous) and Ks (synonymous) substitution rates were calculated using TBtools, revealing that all analyzed gene pairs exhibited Ka/Ks ratios <1 (**Table 2**), indicative of purifying selection during evolution. The divergence times for these duplication events ranged from 10.23 to 54.13 million years ago (MYA), as estimated through molecular clock analysis.

**Table 2:** Evolutionary selection pressure analysis of *BrAPYs*.

Duplicated genes	Ka	Ks	Ka_Ks	Duplication time (MYA)
BrAPY1-4/BrAPY1-1	0.082419049	0.748998548	0.110038996	29.97
BrAPY1-1/BrAPY1-3	0.072832792	0.693237127	0.105061874	23.11
BrAPY1-2/BrAPY1-4	0.265054968	1.037151124	0.255560604	34.58
BrAPY1-2/BrAPY1-1	0.032966934	0.306983450	0.107389938	10.23
BrAPY4-1/BrAPY6-1	0.215596606	0.692511699	0.311325579	23.08
BrAPY4-1/BrAPY6-2	0.420633664	0.855280684	0.491807744	28.51
BrAPY6-1/BrAPY7-2	0.557340630	1.623852197	0.343221280	54.13
BrAPY7-1/BrAPY7-2	0.071200385	0.533142453	0.133548518	17.78

### 3.5 Cis-regulatory element analysis

**Figure 4:**
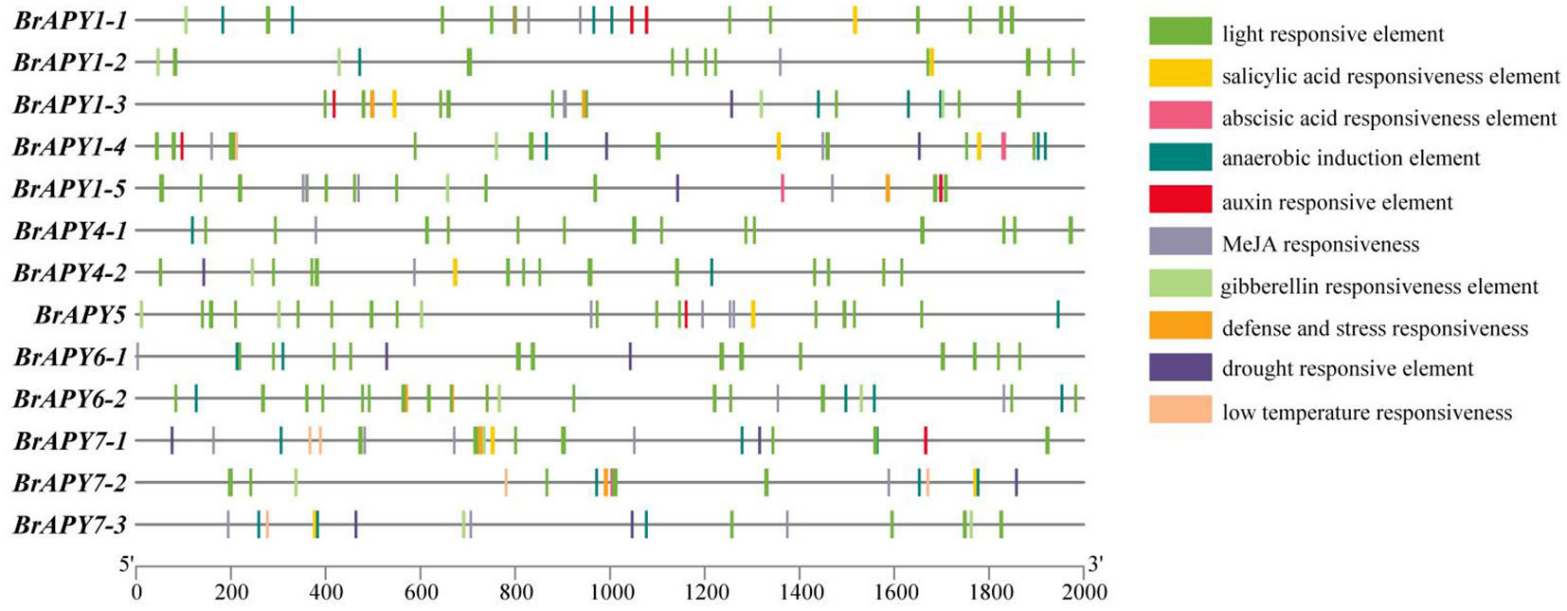
Cis-regulatory elements in *BrAPY* promoter regions. Color-coded boxes represent distinct functional elements located within 2 kb upstream of transcriptional start sites.

Promoter analysis of the 2-kb upstream regions revealed four major categories of cis-regulatory elements in *BrAPYs* (**Figure 4**): (1) hormone responsive elements (MeJA-, salicylic acid-, gibberellin-, abscisic acid-, auxin-responsive), (2) abiotic stress responsive elements (hypoxia-, low temperature-, drought-, and defense-related), (3) light-responsive elements (CTT-Motif, TCC-motif, GT1-motif, G-box, BOX 4), and (4) growth/development-associated elements (AT-rich, zein metabolism, O2-site).

*BrAPY1–4* contained the highest density of these predicted cis-regulatory elements, while *BrAPY1–2* and *BrAPY7–2* showed the simplest profiles (**Figure 4**). All *BrAPYs* promoters harbored two conserved methyl jasmonate-responsive motifs (CGTCA-motif and TGACG-motif), and most contained antioxidant response elements (ARE), which may be involved in oxidative stress adaptation.

### 3.6 Phylogenetic and gene duplication analysis

**Figure 5:**
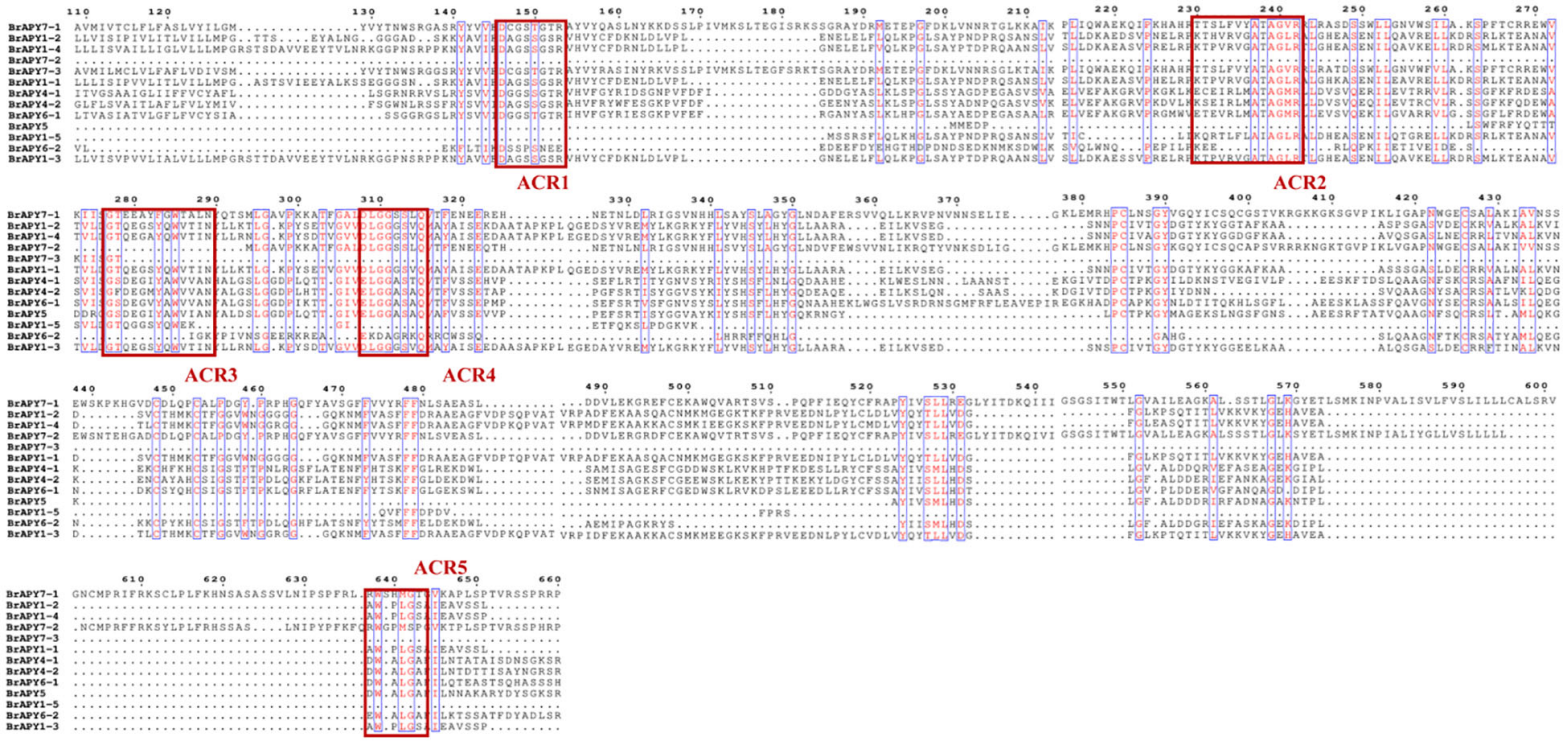
13 BrAPYs amino acid sequence alignment analysis. The red box indicates the location of ACR.

The comparison analysis of 13 BrAPY amino acid sequences shows that the APY gene sequences in Chinese cabbage are overall similar to those of *Arabidopsis thaliana*, and also contain 5 conserved regions of Apyrase (ACR1 to ACR5), which are consistent with the characteristics of this type of gene (**Figure 5**).

**Figure 6:**
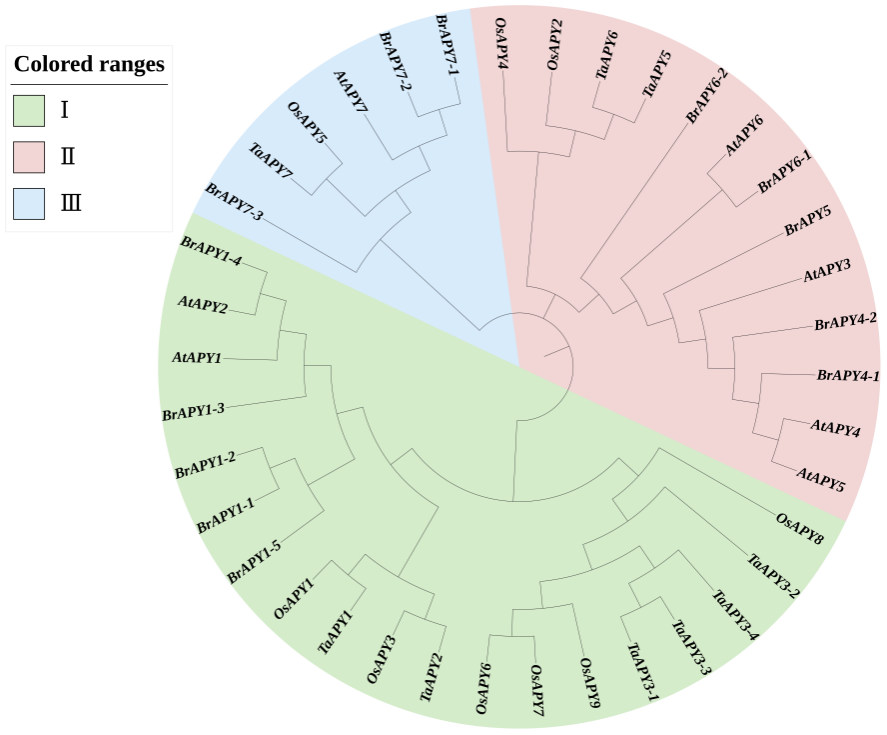
Phylogenetic relationships of APY proteins across four plant species. Genes were divided into three different groups, each marked with a different color.

A Neighbor-Joining phylogenetic tree was constructed using APY protein sequences from *Arabidopsis thaliana*, wheat, rice, and *B.rapa* (**Figure 6**). The *APY* genes were classified into three distinct clades: Group I consists of 5 *BrAPY* genes; Group II consists of 5 *BrAPY* genes; and Group III consists of 3 *BrAPY* genes, representing the smallest phylogenetic cluster with only three members.

### 3.7 Synteny analysis

**Figure 7:**
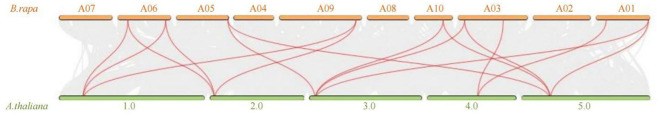
Inter-genomic synteny of *APY* genes. Gray background lines denote whole-genome collinear blocks, while red lines highlight conserved APY homologs between *B.rapa* and *Arabidopsis thaliana*.

Comparative genomic analysis revealed conserved syntenic relationships between *B.rapa* and *Arabidopsis thaliana APY* genes (**Figure 7**). Specifically, *BrAPYs* in Group I exhibited one-to-one orthology with *AtAPY1/AtAPY2*, Group II members predominantly showed homology to *AtAPY4/AtAPY6*, while Group III *BrAPYs* demonstrated evolutionary conservation with *AtAPY7*.

### 3.8 Tertiary structure prediction

**Figure 8:**
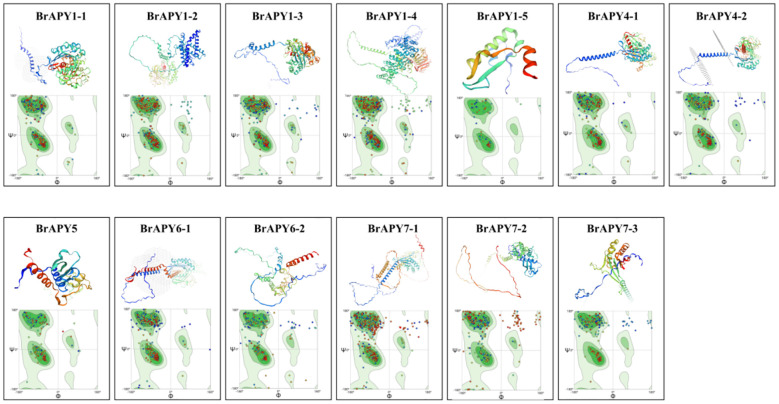
The three-dimensional (3D) structure of APY proteins in *B.rapa*. In 3D structures, helices constitute the spirals, beta-pleated sheets manifest as broad strips with arrowheads, and coils are represented by narrow loops. Ramachandran plots depict dark green, and light green correspond to conformations that are highly favored, with a Delta value greater than or equal to − 2. The color white combined with a black grid pattern is indicative of favored conformations within a range of − 2 to − 4 for the variable Delta.

The three-dimensional structure prediction of BrAPY proteins was performed on the SWISS-MODEL platform using their protein sequences (**Figure 8**). The sequence identity among the 13 BrAPYs ranged from 71.29% to 100%, indicating a relatively high modeling quality (screening **Supplementary Table 4**). Analysis of the Ramachandran plot revealed that over 90% of the residues in the APY proteins exhibited favorable conformations, thereby confirming the accuracy of the backbone dihedral angles of the studied proteins (screening **Supplementary Table 5**). These findings suggest that the predicted protein structures are stable and of high quality (Samra et al., 2024). Furthermore, the results of tertiary structure modeling indicate that the three-dimensional arrangements of most BrAPY proteins are highly similar. Based on these observations, it can be reasonably speculated that BrAPY proteins may possess similar physiological regulatory functions (Yuan et al., 2024).

### 3.9 Tissue-specific expression patterns of *BrAPY* genes in *B.rapa*

**Figure 9:**
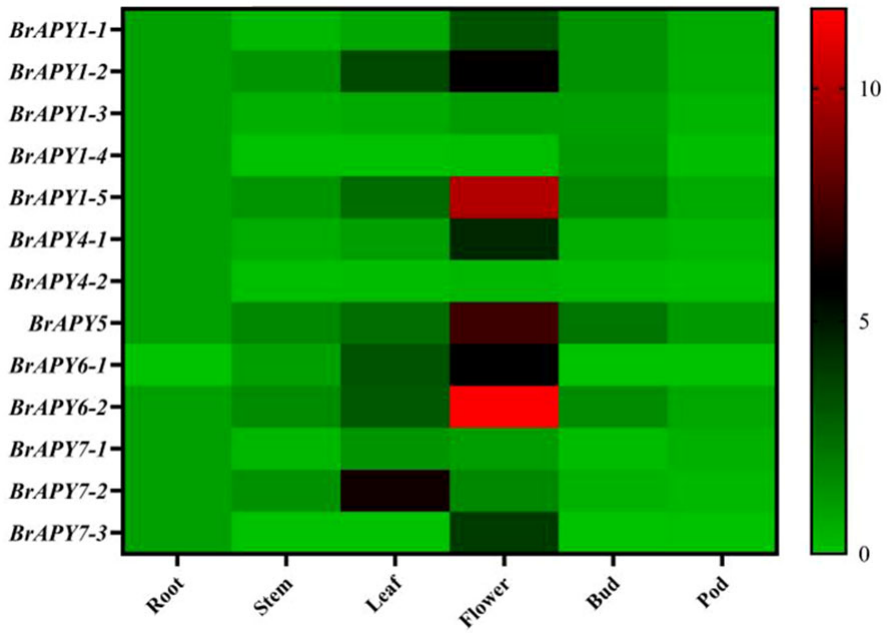
Heatmap of the tissue-specific expression patterns of *BrAPY* genes in various organs. Colors in the graph indicate the level of expression, light colors represent low expression levels, while red represents high expression levels. The data were all log-transformed to enhance contrast.

To further elucidate the biological roles of *BrAPY* genes, their tissue-specific expression patterns were examined across six major organs: roots, stems, leaves, flowers, buds, and pods. qRT-PCR analysis revealed that *BrAPY* genes exhibited preferential expression in flowers (**Figure 9**). Notably, *BrAPY1-5*, *BrAPY5-2*, and *BrAPY5* displayed peak expression levels in flowers, with transcript abundances significantly higher than in other tissues. These findings suggest that these genes may play critical roles in reproductive processes, such as floral development and seed formation in *B.rapa*.

### 3.10 Stress-responsive expression patterns of *BrAPY* genes

**Figure 10:**
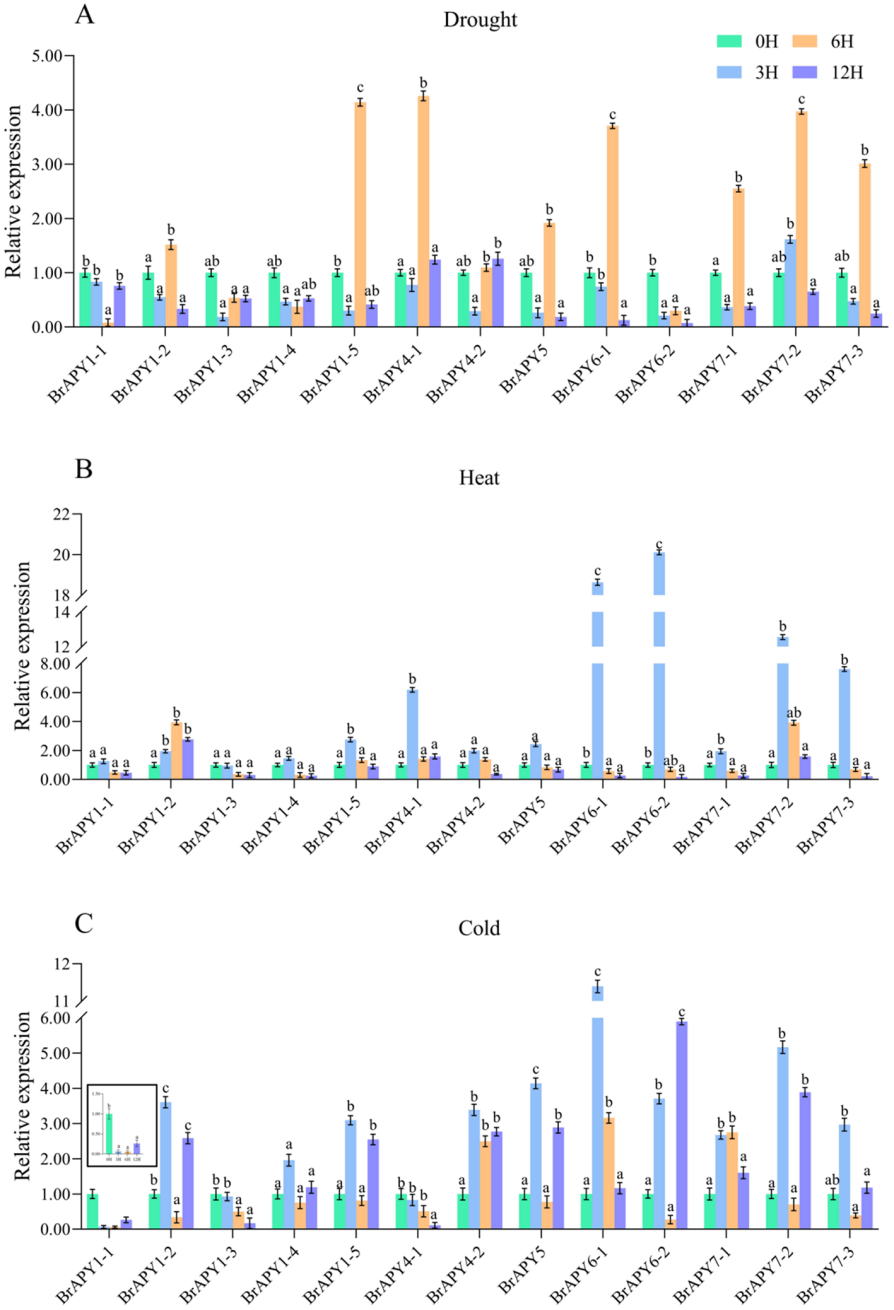
Temporal expression profiles of *BrAPYs* under drought **(A)**, High-temperature **(B)**, and low-temperature stress **(C)**. Transcript levels were quantified by qRT-PCR in *B.rapa* leaves at 0 (baseline), 3, 6, and 12 hpt. Data represent mean ± SEM (n=3 biological replicates). Letters denote significant differences (p<0.05) between timepoints based on one-way ANOVA with Fisher’s LSD *post-hoc* test.

The temporal expression dynamics of *BrAPY* genes under abiotic stresses were systematically analyzed using qRT-PCR (**Figure 10**). Under drought stress, the majority of *BrAPY* genes exhibited peak expression levels at 6 hours post-treatment (hpt), with *BrAPY1-5*, *BrAPY4-1*, *BrAPY6-1*, *BrAPY7-2*, and *BrAPY7–3* showing ≥2.0-fold increases relative to their respective baseline levels. By 12 hpt, however, all genes had either returned to baseline levels or were significantly downregulated (**Figure 10A**).

In contrast, high-temperature treatment induced more rapid transcriptional responses in most *BrAPYs*, with maximum expression levels occurring at 3 hpt. Notably, *BrAPY6–2* demonstrated exceptional sensitivity (20.0-fold induction, p<0.001), followed by *BrAPY6-1* (about 18-fold, p<0.001). After this time point, their expression levels gradually declined. Nevertheless, some genes, such as *BrAPY1-2*, *BrAPY4-1*, and *BrAPY7-2*, maintained higher-than-baseline expression levels at 12 hpt (**Figure 10B**), suggesting sustained heat-stress adaptation mechanisms.

Under low-temperature exposure, most *BrAPY* genes also exhibited a rapid transcriptional response at 3 hpt. However, unlike the pattern observed under high-temperature stress, low-temperature treatment elicited a biphasic regulatory pattern, with most genes maintaining >2-fold expression levels at 12 hpt (**Figure 10C**). This prolonged activation phase suggests potential involvement in cold acclimation processes.

In conclusion, comparative analysis revealed stronger transcriptional responses to thermal stresses compared to drought, indicating temperature-responsive specialization within the *BrAPY* family (**Figure 10**). This differential responsiveness may reflect specialized roles in temperature-sensitive physiological processes, such as cold acclimation or thermotolerance.

## 4 Discussion

Since its identification, extracellular ATP (eATP) has been recognized as a universal signaling molecule (Tanaka et al., 2010). In plants, eATP coordinates diverse responses through APY-mediated regulation (Deng et al., 2015). While *APYs* have been characterized in model species and cereals (Roux and Steinebrunner, 2007; Choi et al., 2014), they remained unstudied in *B. rapa.*

Our genome-wide analysis systematically identified 13 *APY* genes in *B.rapa*, all containing the conserved GDA1/CD39 domains. Phylogenetic reconstruction using neighbor-joining method revealed three evolutionarily distinct clades (**Figure 5**). Notably, *BrAPY1-5* (Group I), *BrAPY5* and *BrAPY6-2* (Group II) formed divergent branches within their respective clades, suggesting potential functional divergence. Chromosomal mapping localized these atypical genes to distinct genomic regions: *BrAPY5* on chromosome A7 and *BrAPY6–2* on chromosome A9, contrasting with the clustering of other Group II members on chromosome A6 (**Figure 1**). Evolutionary analysis identified eight paralogous gene pairs, predominantly under purifying selection (Ka/Ks < 1), indicating strong functional constraints during *Brassica* evolution. Structural conservation patterns emerged through exon-intron analysis, with *BrAPY1–5* displaying a unique architecture characterized by additional exons (**Figure 2B**). Promoter analysis predicted the presence of multiple hormone-responsive cis-elements (**Figure 4**), suggesting their potential roles in transcriptional regulation.

Spatiotemporal expression profiling revealed preferential expression of *BrAPY* genes in metabolically active tissues (stems, leaves, flowers), consistent with their hypothesized roles in energy metabolism (Wang et al., 2024). Plants also exhibit genetic-level responses to stress. When exposed to stressful conditions, they activate signal transduction pathways, which ultimately result in gene expression. This process enables plants to develop resistance mechanisms against various stresses (Rehaman et al., 2025).In many plants, the *APY* family has been shown to play a critical role in responding to abiotic stress (Chowdhury et al., 2023). The expression pattern of the *BrAPY* gene was analyzed by qRT-PCR to elucidate their roles in various stress responses. Stress-response assays demonstrated significant transcriptional induction under thermal and drought stresses, with distinct kinetic patterns: rapid peaks at 3 hpt under temperature extremes versus delayed maxima at 6 hpt under drought (**Figure 10**). The observed biphasic expression patterns (initial induction, followed by transient suppression, and subsequent secondary activation) suggest the involvement of complex regulatory networks in coordinating stress adaptation (Qiao et al., 2020). Notably, *BrAPY6–2* exhibited sustained upregulation (>5.9-fold at 12 hpt) under cold stress, potentially facilitating long-term acclimation (Zhang et al., 2024).

These findings suggest that *BrAPYs* may function as critical mediators of abiotic stress responses in *B.rapa*, with clade-specific expression dynamics hinting at functional specialization during Brassica evolution. The differential stress responsiveness between thermal and drought treatments implies that temperature could be a primary evolutionary driver for *APY* diversification.

## 5 Conclusions

This study systematically identified and characterized 13 *APY* genes in the *B. rapa* genome. They were phylogenetically classified into three groups, show an uneven chromosomal distribution, and possess promoters enriched in stress- and hormone-responsive elements. Expression analysis revealed tissue-specific patterns and distinct temporal responses to drought, heat, and cold stress, with generally stronger induction under temperature extremes. These findings highlight the functional diversification of the *APY* family in Chinese cabbage and provide a foundation for future research into their roles in stress adaptation, ultimately supporting the development of more resilient cultivars.

## Data Availability

The datasets presented in this study can be found in online repositories. The names of the repository/repositories and accession number(s) can be found in the article/**Supplementary Material**.

## References

[B1] Anderson R. J. Weng Z. Campbell R. K. Jiang X. (2005). Main-chain conformational tendencies of amino acids. Proteins 60, 679–689. doi: 10.1002/prot.20530 16021632

[B2] Bailey T. L. Boden M. Buske F. A. Frith M. Grant C. E. Clementi L. . (2009). MEME SUITE: tools for motif discovery and searching. Nucleic Acids Res. 37, W202–W208. doi: 10.1093/nar/gkp335 19458158 PMC2703892

[B3] Chen C. Wu Y. Li J. Wang X. Zeng Z. Xu J. . (2023). TBtools-II: A "one for all, all for one" bioinformatics platform for biological big-data mining. Mol. Plant 16, 1733–1742. doi: 10.1016/j.molp.2023.09.010 37740491

[B4] Chen H. Wang T. He X. Cai X. Lin R. Liang J. . (2022). BRAD V3.0: an upgraded Brassicaceae database. Nucleic Acids Res. 50, D1432–D1441. doi: 10.1093/nar/gkab1057 34755871 PMC8728314

[B5] Chiu T. Y. Lao J. Manalansan B. Loqué D. Roux S. J. Heazlewood J. L. (2015). Biochemical characterization of Arabidopsis APYRASE family reveals their roles in regulating endomembrane NDP/NMP homoeostasis. Biochem. J. 472, 43–54. doi: 10.1042/BJ20150235 26338998

[B6] Chivasa S. Ndimba B. K. Simon W. J. Lindsey K. Slabas A. R. (2005). Extracellular ATP functions as an endogenous external metabolite regulating plant cell viability. Plant Cell 17, 3019–3034. doi: 10.1105/tpc.105.036806 16199612 PMC1276027

[B7] Choi J. Tanaka K. Liang Y. Cao Y. Lee S. Y. Stacey G. (2014). Extracellular ATP, a danger signal, is recognized by DORN1 in Arabidopsis. Biochem. J. 463, 429–437. doi: 10.1042/BJ20140666 25301072

[B8] Chowdhury A. T. Hasan M. N. Bhuiyan F. H. Islam M. Q. Nayon M. R. W. Rahaman M. M. . (2023). Identification, characterization of Apyrase (APY) gene family in rice (Oryza sativa) and analysis of the expression pattern under various stress conditions. PloS One 18, e0273592. doi: 10.1371/journal.pone.0273592 37163561 PMC10171694

[B9] Clark G. Brown K. A. Tripathy M. K. Roux S. J. (2021). Recent advances clarifying the structure and function of plant apyrases (nucleoside triphosphate diphosphohydrolases). Int. J. Mol. Sci. 22, 3283. doi: 10.3390/ijms22063283 33807069 PMC8004787

[B10] Clark G. Roux S. J. (2018). Role of Ca2+ in mediating plant responses to extracellular ATP and ADP. Int. J. Mol. Sci. 19, 3590. doi: 10.3390/ijms19113590 30441766 PMC6274673

[B11] Cohn J. R. Uhm T. Ramu S. Nam Y. W. Kim D. J. Penmetsa R. V. . (2001). Differential regulation of a family of apyrase genes from Medicago truncatula. Plant Physiol. 125, 2104–2119. doi: 10.1104/pp.125.4.2104 11299390 PMC88866

[B12] Deng S. Sun J. Zhao R. Ding M. Zhang Y. Sun Y. . (2015). Populus euphratica APYRASE2 enhances cold tolerance by modulating vesicular trafficking and extracellular ATP in Arabidopsis plants. Plant Physiol. 169, 530–548. doi: 10.1104/pp.15.00581 26224801 PMC4577398

[B13] He Z. Zhang J. Jia H. Zhang S. Sun X. Nishawy E. . (2024). Correction to: Genome-wide identification and analyses of ZmAPY genes reveal their roles involved in maize development and abiotic stress responses. Mol. Breeding: New Strategies Plant Improvement 44, 44. doi: 10.1007/s11032-024-01482-9 38745883 PMC11091030

[B14] Hu B. Jin J. Guo A. Y. Zhang H. Luo J. Gao G. (2015). GSDS 2.0: an upgraded gene feature visualization server. Bioinf. (Oxford England) 31, 1296–1297. doi: 10.1093/bioinformatics/btu817 25504850 PMC4393523

[B15] Kayum M. A. Jung H. J. Park J. I. Ahmed N. U. Saha G. Yang T. J. . (2015). Identification and expression analysis of WRKY family genes under biotic and abiotic stresses in Brassica rapa. Mol. Genet. Genomics: MGG 290, 79–95. doi: 10.1007/s00438-014-0898-1 25149146

[B16] Koch M. A. Haubold B. Mitchell-Olds T. (2000). Comparative evolutionary analysis of chalcone synthase and alcohol dehydrogenase loci in Arabidopsis, Arabis, and related genera (Brassicaceae). Mol. Biol. Evol. 17, 1483–1498. doi: 10.1093/oxfordjournals.molbev.a026248 11018155

[B17] Lim M. H. Wu J. Yao J. Gallardo I. F. Dugger J. W. Webb L. J. . (2014). Apyrase suppression raises extracellular ATP levels and induces gene expression and cell wall changes characteristic of stress responses. Plant Physiol. 164, 2054–2067. doi: 10.1104/pp.113.233429 24550243 PMC3982762

[B18] Liu W. Ni J. Shah F. A. Ye K. Hu H. Wang Q. . (2019). Genome-wide identification, characterization and expression pattern analysis of APYRASE family members in response to abiotic and biotic stresses in wheat. PeerJ 7, e7622. doi: 10.7717/peerj.7622 31565565 PMC6744936

[B19] Mistry J. Chuguransky S. Williams L. Qureshi M. Salazar G. A. Sonnhammer E. L. L. . (2021). Pfam: The protein families database in 2021. Nucleic Acids Res. 49, D412–D419. doi: 10.1093/nar/gkaa913 33125078 PMC7779014

[B20] Mohammad-Sidik A. Sun J. Shin R. Song Z. Ning Y. Matthus E. . (2021). Annexin 1 is a component of eATP-induced cytosolic calcium elevation in Arabidopsis thaliana roots. Int. J. Mol. Sci. 22, 494. doi: 10.3390/ijms22020494 33419052 PMC7825420

[B21] Paysan-Lafosse T. Blum M. Chuguransky S. Grego T. Pinto B. L. Salazar G. A. . (2023). InterPro in 2022. Nucleic Acids Res. 51, D418–D427. doi: 10.1093/nar/gkac993 36350672 PMC9825450

[B22] Qiao Y. Gao X. Liu Z. Wu Y. Hu L. Yu J. (2020). Genome-wide identification and analysis of SRO gene family in Chinese cabbage (Brassica rapa L.). Plants (Basel Switzerland) 9, 1235. doi: 10.3390/plants9091235 32962109 PMC7569827

[B23] Quan J. Zheng W. Tan J. Li Z. Wu M. Hong S. B. . (2022). Glutamic acid and poly-γ-glutamic acid enhanced the heat resistance of Chinese cabbage (Brassica rapa L. ssp. pekinensis) by improving carotenoid biosynthesis, photosynthesis, and ROS signaling. Int. J. Mol. Sci. 23, 11671. doi: 10.3390/ijms231911671 36232971 PMC9570168

[B24] Rehaman A. Khan S. Rawat B. Gaira K. S. Asgher M. Semwal P. . (2025). Mechanistic insights into plant drought tolerance: A multi-level perspective. J. Crop Health 77, 53. doi: 10.1007/s10343-025-01115-x 30311153

[B25] Riewe D. Grosman L. Fernie A. R. Wucke C. Geigenberger P. (2008). The potato-specific apyrase is apoplastically localized and has influence on gene expression, growth, and development. Plant Physiol. 147, 1092–1109. doi: 10.1104/pp.108.117564 18480378 PMC2442552

[B26] Robson S. C. Sévigny J. Zimmermann H. (2006). The E-NTPDase family of ectonucleotidases: Structure function relationships and pathophysiological significance. Purinergic Signalling 2, 409–430. doi: 10.1007/s11302-006-9003-5 18404480 PMC2254478

[B27] Roux S. J. Steinebrunner I. (2007). Extracellular ATP: an unexpected role as a signaler in plants. Trends Plant Sci. 12, 522–527. doi: 10.1016/j.tplants.2007.09.003 17928260

[B28] Samra I. Nazia R. Safeena I. Muhammad Z. F. K. Muhammad R. K. (2024). Genome-wide identification and expression profiling of Pseudo-Response Regulator (PRR) gene family in tomato. Environ. Exp. Bot. 220, 105683. doi: 10.1016/j.envexpbot.2024.105683 38826717

[B29] Sharif Y. Mamadou G. Yang Q. Cai T. Zhuang Y. Chen K. . (2023). Genome-wide investigation of apyrase (APY) genes in peanut (Arachis hypogaea L.) and functional characterization of a pod-abundant expression promoter AhAPY2-1p. Int. J. Mol. Sci. 24, 4622. doi: 10.3390/ijms24054622 36902052 PMC10003104

[B30] Steinebrunner I. Wu J. Sun Y. Corbett A. Roux S. J. (2003). Disruption of apyrases inhibits pollen germination in Arabidopsis. Plant Physiol. 131, 1638–1647. doi: 10.1104/pp.102.014308 12692323 PMC166920

[B31] Swarbreck D. Wilks C. Lamesch P. Berardini T. Z. Garcia-Hernandez M. Foerster H. . (2008). The Arabidopsis Information Resource (TAIR): gene structure and function annotation. Nucleic Acids Res. 36, D1009–D1014. doi: 10.1093/nar/gkm965 17986450 PMC2238962

[B32] Tanaka K. Gilroy S. Jones A. M. Stacey G. (2010). Extracellular ATP signaling in plants. Trends Cell Biol. 20, 601–608. doi: 10.1016/j.tcb.2010.07.005 20817461 PMC4864069

[B33] Wang L. Zhao Z. Li H. Pei D. Ma Q. Huang Z. . (2024). Genome-wide identification and molecular evolutionary history of the Whirly family genes in Brassica napus. Plants (Basel Switzerland) 13, 2243. doi: 10.3390/plants13162243 39204679 PMC11359715

[B34] Waterhouse A. Bertoni M. Bienert S. Studer G. Tauriello G. Gumienny R. . (2018). SWISS-MODEL: homology modelling of protein structures and complexes. Nucleic Acids Res. 46, W296–W303. doi: 10.1093/nar/gky427 29788355 PMC6030848

[B35] Wu J. Steinebrunner I. Sun Y. Butterfield T. Torres J. Arnold D. . (2007). Apyrases (nucleoside triphosphate-diphosphohydrolases) play a key role in growth control in Arabidopsis. Plant Physiol. 144, 961–975. doi: 10.1104/pp.107.097568 17434987 PMC1914212

[B36] Yuan Y. Wang L. Zhao Q. Liu C. Fu X. Li X. . (2024). Genome-wide identification, expression analysis and functional study of DELLA genes in Chinese cabbage (Brassica rapa L. ssp. pekinensis). Front. Bioscience (Landmark Edition) 29, 198. doi: 10.31083/j.fbl2905198 38812324

[B37] Zhang M. Wu X. Chen L. Yang L. Cui X. Cao Y. (2024). The RopGEF gene family and their potential roles in responses to abiotic stress in Brassica rapa. Int. J. Mol. Sci. 25, 3541. doi: 10.3390/ijms25063541 38542514 PMC10971340

[B38] Zimmermann H. (2021). History of ectonucleotidases and their role in purinergic signaling. Biochem. Pharmacol. 187, 114322. doi: 10.1016/j.bcp.2020.114322 33161020

